# Pulmonary Delivery of Inhalable Sustained Release Nanocomposites Microparticles Encapsulating Osimertinib for Non-Small Cell Lung Cancer Therapy

**DOI:** 10.3390/pharmaceutics18010134

**Published:** 2026-01-21

**Authors:** Iman M. Alfagih, Alanood Almurshedi, Basmah Aldosari, Bushra Alquadeib, Baraa Hajjar, Hafsa Elwali, Hadeel ALtukhaim, Eman Alzahrani, Sara Alhumaidan, Ghaida Alharbi

**Affiliations:** Department of Pharmaceutics, College of Pharmacy, King Saud University, Riyadh 11451, Saudi Arabia

**Keywords:** lung cancer, osimertinib, nanocomposite microparticles, dry powder inhalation, pulmonary drug delivery

## Abstract

**Background/Objective:** Osimertinib (OSI) is a third-generation tyrosine kinase inhibitor approved for non-small cell lung cancer (NSCLC) therapy. OSI is administered orally; this route limits the amount of OSI reaching the tumor in the lungs and is associated with serious systemic toxicity. This study aimed to develop a dry powder inhalable formulation to provide tumor-targeted delivery and minimize systemic toxicity. To the best of our knowledge, this is the first study to prepare and evaluate a dry powder inhalation formulation of OSI. **Methods:** Chitosan-coated PLGA nanoparticles (PLGA-C NPs) encapsulating OSI were prepared using a single emulsion-solvent evaporation technique. PLGA-C NPs were assembled into respirable nanocomposite microparticles (NCMPs) via spray drying with L-leucine as a carrier. PLGA-C NPs were characterized for particle size, zeta-potential, encapsulation efficiency, and in vitro efficacy in A-549 cell line. NCMPs were evaluated for solid-state properties, aerosolization performance, stability and in vitro release. **Results:** PLGA-C NPs exhibited a particle size of 145.18 ± 3.0 nm, high encapsulation efficiency and a positive zeta potential. In vitro studies demonstrated a 3.6-fold reduction in IC50 compared to free OSI, superior antimigratory effects and enhanced cell cycle arrest. Solid-state characterization of NCMPs demonstrated drug encapsulation in the polymer without chemical interaction. NCMPs exhibited excellent aerosolization (mass median aerodynamic diameter of 1.09 ± 0.23 μm, fine particle fraction of 73.48 ± 8.6%) and sustained drug release (61.76 ± 3.9% at 24 h). Stability studies confirmed the physicochemical stability integrity. **Conclusions:** These findings suggest that this novel dry powder inhalable OSI formulation may improve therapeutic outcomes while reducing systemic toxicity.

## 1. Introduction

The second-most prevalent cancer in both men and women is lung cancer, both small cell and non-small cell lung cancer (NSCLC). Lung cancer is the most common cause of cancer-related mortality among men and women, representing over 25% of total cancer deaths [1]. With a prevalence of up to 85% among lung cancer cases, NSCLC is linked to a five-year survival rate of under 15% [2].

Traditional lung cancer therapy includes chemotherapy, surgery, and radiotherapy. Most of these traditional therapies suffer from off-target effects [3]. Targeted therapy, which includes gene expression modulators and immunotherapy, has been widely studied in recent years. One of the main driving genes of NSCLC is the epidermal growth factor receptor (EGFR) [4]. Tyrosine kinase inhibitors (TKIs) are chemicals that bind to the EGFR and have been shown to have a higher response rate in individuals with sensitizing EGFR mutations than standard chemotherapy [5]. However, resistance to TKIs therapy is an unavoidable outcome. Using first- and second-generation TKIs, for instance, erlotinib and gefitinib, leads to secondary resistance mechanisms [6].

Osimertinib (OSI) is a third-generation TKIs that is FDA-approved as a first-line therapy for EGFR T790M mutation-positive NSCLC [7]. OSI is administered orally as a film-coated tablet, which limits the amount of drug reaching the tumor in the lungs. Serious systemic toxicities like diarrhea, skin rashes, mucositis and renal symptoms are frequently reported, which prevents its ongoing and widespread usage [8,9]. Patients treated with OSI, on the other hand, eventually develop acquired resistance, preventing it from providing long-term benefits to patients [10,11]. As a result, developing effective approaches to overcome the conventional dosage form limitations will address a significant clinical challenge while also benefiting patients by increasing their survival time.

Pulmonary delivery of anticancer drug-loaded nanoparticles offers the passive targeting of tumors. Inhalations provide localized delivery to the lungs, achieving high drug concentrations at the primary tumor site while minimizing exposure to healthy tissues. Compared to intravenous or oral administration, inhalation produces significantly lower systemic drug levels, reducing adverse effects. Furthermore, dry powder inhalation offers many advantages; for instance, it eliminates cold-chain requirements and improves physical and chemical stability of solid formulations over liquid formulation [12,13,14,15].

PLGA, a biodegradable and biocompatible polymer with FDA approval, has been utilized extensively for drug delivery. Small-molecule drugs and biomacromolecules can be packaged in PLGA nanoparticles to increase effectiveness and prevent degradation. PLGA nanoparticles, however, lack attachment to body cells and are typically negatively charged. The PLGA nanoparticles were coated using chitosan to overcome these limitations. The amino group of chitosan confers a positive charge on the nanoparticles when chitosan is used to coat the PLGA nanoparticles, which improves membrane permeability and facilitates efficient intracellular drug uptake. Moreover, chitosan contributes to improved nanoparticles adhesion, retention, and diffusion. Furthermore, chitosan has anti-cancer activity by inhibiting cell growth via the apoptotic pathway [16].

However, nanoparticles cannot be deposited directly into the lungs because their nanometer size range (<1 µm) prevents them from settling in the alveoli and being breathed out. Moreover, large particles (>5 µm) usually deposit in the oropharynx from which they are easily cleared. Only particles with a diameter of 1–5 µm can reach the alveoli and settle within the lower region of the lung. Thus, to achieve an aerodynamic size of 1–5 μm, nanoparticles may be integrated into microparticles, resulting in nanocomposite microparticles (NCMPs). Upon inhalation in a dry powder form, these particles achieve the ideal size for lung deposition and disintegrate in the pulmonary lining fluid, liberating the nanocomposites from the carrier microparticles [17,18]. Because of their unique sizes and fluidity, dry powder inhalation of NCMPs offers significant benefits. These NCMPs characteristics provide the drug to the action site and nanoparticles (less than 260 nm) can significantly minimize phagocytosis or mucociliary clearance in the process. NCMPs integrated the advantages of microparticles and nanoparticles for pulmonary drug delivery [19,20].

Recently, pulmonary delivery of OSI has primarily been investigated using liquid-based formulations [21,22,23,24,25]. Despite these advancements, the limited stability of liquid formulations—lasting only a few weeks—remains a significant challenge. Therefore, the present study aims to develop a dry powder inhalation formulation of OSI to potentially improve its efficacy, achieve a sustained drug release to improve patient adherence by reducing dosing frequency and improve long-term stability of the formulation. To the best of our knowledge, this is the first study to prepare and evaluate a dry powder inhalation formulation of OSI.

## 2. Materials and Methods

### 2.1. Materials

Osimertinib was obtained from Selleck Chemicals (Shanghai, China). Acid-terminated poly (lactic acid-co-glycolic acid) (PLGA, 50:50; MW 7–17 kDa) was purchased from Liaoning Kuke Biotechnology Co., Ltd. (Fuxin City, China). Low molecular weight chitosan (MW 50,000–190,000; degree of deacetylation 75–85%) was obtained from Merck KGaA (Darmstadt, Germany). Poly (vinyl alcohol) (PVA, MW 13–23 kDa, 87–89% hydrolyzed) and L-leucine were purchased from BioUltra, Sigma-Aldrich, Gillingham, UK. Acetonitrile and dichloromethane were purchased from Fisher Scientific (Chatham, UK). The A549 lung cancer cell line (ATCC CRM-CCL-185^TM^ (Adherent)) was obtained from American Type Culture Collection (ATCC, Manassas, VA, USA). Dulbecco’s Modified Eagle medium (DMEM) supplemented with 10% Fetal Bovine serum (FBS) were purchased from ThermoFisher Scientific, Waltham, MA, USA.

### 2.2. Preparation of Nanoparticles

Emulsification-solvent evaporation method, with slight modification, was employed to formulate OSI-loaded PLGA nanoparticles (PLGA-NPs) [16]. Briefly, OSI (2 mg) was first dissolved in ethanol, which was then added to an organic solution of PLGA (50 mg PLGA in dichloromethane) and probe sonicated by means of a probe sonicator (VC X 500 Vibra-CellTM, 13 mm probe, Sonics & Materials, Inc., Newtown, CT, USA) for 120 s (10 s on-off cycle, 40% amplitude). This primary solution was subjected to probe sonication for 240 s (10 s on-off cycle) at 40% amplitude in an aqueous solution containing polyvinyl alcohol (PVA, 1% *w*/*v*) as a stabilizer, at a volume ratio of 1:10 (OSI–PLGA mixture: PVA solution). The resulting emulsion was stirred for 2 h at room temperature to allow organic solvent evaporation. PLGA-NPs were separated from the aqueous suspension medium by centrifugation (Sigma 3–30k; SIGMA Laborzentrifugen GmbH, Osterode am Harz, Germany) at 40,000 rcf for 15 min (4 °C) followed by two washes with water using the same centrifugation conditions. To prepare the chitosan-coated nanoparticles (PLGA-C NPs) the same procedure was followed with a slight modification: chitosan (1% *w*/*v*) was added to the PVA aqueous solution containing a 1% *v*/*v* acetic acid.

### 2.3. Preparation of Nanocomposites Microparticles

To convert the recovered nanoparticles into dry powder for inhalation, the nanoparticles were suspended in an aqueous solution of L-leucine at 1:1.5 *w*/*w* ratio (PLGA:L-leucine). The suspension of nanoparticles and L-leucine was spray-dried employing a Büchi B-290 mini spray dryer (Büchi Labortechnik, Flawil, Switzerland), equipped with a 0.7 mm two-fluid nozzle and a high-efficiency cyclone, to produce nanocomposite nanoparticles (NCMPs). The process parameters for spray-drying included: feed concentration of 12.5 mg/mL, 10% feed rate, 100% aspirator capacity, 400 L/h atomizing air flow, 100 °C inlet temperature and outlet temperature between 42 and 46 °C [20]. The NCMPs powder was collected from the high efficiency cyclone and stored at room temperature in a desiccator until further use.

### 2.4. Characterization of Nanoparticles

#### 2.4.1. Particle Size, Polydispersity Index and Zeta Potential

The mean particle size, polydispersity index (PDI), and zeta potential were determined by dynamic light scattering (DLS) using Zetasizer Nano ZS (Malvern Instruments Limited, Worcestershire, UK). The measurements were conducted at ambient temperature (25 °C) by diluting an aliquot of formulation with deionized water (n = 3).

#### 2.4.2. Morphology

The morphology of nanoparticles was observed by transmission electron microscope (TEM, JEOL 1400 PLUS, Tokyo, Japan). The sample was placed on the grids and allowed to air dry at ambient temperature for 10 min; grids were formvar/carbon on 200 mesh copper (purchased from Calibre Scientific, Sheffield, UK).

#### 2.4.3. High-Performance Liquid Chromatography Assay

A Waters high-performance liquid chromatography (HPLC) system (Waters Corporation, Milford, MA, USA) was employed to quantify OSI provided with Waters Symmetry C18 column (4 μm, 3.9 × 250 mm). HPLC chromatographic conditions included a mobile phase of 0.1% (*v*/*v*) orthophosphoric acid and acetonitrile at 20:80 (*v*/*v*) ratio, a flow rate of 0.5 mL/min, with a detection at 276 nm and at 40 °C. The retention time for OSI was determined to be 1.731 min [26].

#### 2.4.4. Drug Loading and Encapsulation Efficiency

The amount of OSI loaded into the nanoparticles was quantified indirectly by measuring the residual OSI in the supernatant and wash after centrifugation using HPLC. Peak area was quantified, and free OSI concentrations were derived from the corresponding standard calibration curve. The encapsulation efficiency (%EE) and drug loading (DL) were determined using Equations (1) and (2), respectively (n = 3).

(1) $$\%\mathrm{EE}=\frac{\mathrm{Amount}\;\mathrm{of}\;\mathrm{OSI}\;\mathrm{added}-\mathrm{free}\;\mathrm{amount}\;\mathrm{of}\;\mathrm{OSI}}{\mathrm{Amount}\;\mathrm{of}\;\mathrm{OSI}\;\mathrm{added}}\times 100$$

(2) $$\mathrm{DL}=\frac{\mathrm{Actual}\;\mathrm{amount}\;\mathrm{of}\;\mathrm{OSI}\;\mathrm{encapsulated}}{\mathrm{Total}\;\mathrm{amount}\;\mathrm{of}\;\mathrm{solid}\;\mathrm{added}}$$

The linear range of the standard curve used for determining the concentration of free OSI was 2–20 µg/mL.

### 2.5. Characterization of Nanocomposites Microparticles

#### 2.5.1. Particle Size and Morphology

Scanning electron microscopy (SEM) (FEI—QuantaTM 200 ESEM, Eindhoven, The Netherlands) was employed to examine the morphology of NCMPs. Spray-dried samples were mounted on 13 mm aluminum pin stubs covered with a conductive carbon tab and sputter-coated with palladium (10–15 nm) for 3 min at 25 mA using a EmiTech K 550X Gold Sputter Coater. Additionally, the particle size of NCMPs was evaluated by DLS as detailed in Section 2.4.1.

The nanoparticles recovery from NCMPs was confirmed by re-dispersing an aliquot of the formulation (1 mg) with 4 mL deionized water and vortexed for 30 s at 100 rpm to release the recovered nanoparticles. The mean particle size was determined by DLS as detailed in Section 2.4.1. The redispersibility of dried NCMPs powders in an aqueous medium was assessed using the redispersibility index (RdI). RdI was calculated as the ratio of the mean particle size after reconstitution in water (Sf) to the mean particle size prior to the drying process (Si), using Equation (3):

(3) $$\mathrm{RdI}=\frac{S_{f}}{S_{i}}$$

An RdI value of 1.0 indicates complete redispersion without any change in particle size. Values within the range of 0.7–1.3 were considered acceptable for maintaining nanoparticle integrity after reconstitution [27]. Additionally, the shape of the reconstituted nanoparticles was examined via TEM, as outlined in Section 2.4.2.

#### 2.5.2. Yield of Spray-Dried Formulation

The mass of expected total powder was employed to calculate the percentage yield of NCMPs as a dry powder (n = 3) using Equation (4):

(4) $$\%\mathrm{Yield}=\frac{\mathrm{Weight}\;\mathrm{of}\;\mathrm{NCMPs}\;\mathrm{collected}\;\mathrm{after}\;\mathrm{spray}\;\mathrm{drying}}{\mathrm{Weight}\;\mathrm{of}\;\mathrm{total}\;\mathrm{dry}\;\mathrm{mass}\;\mathrm{used}\;\mathrm{for}\;\mathrm{the}\;\mathrm{preparation}}\times 100$$

#### 2.5.3. Solid-State Characterization

- Differential scanning calorimetry studies

The thermal profiles of OSI, blank PLGA-NCMPS, blank PLGA-C NCMPs, PLGA NCMPs and PLGA-C NCMPs were obtained through a DSC 6000 instrument (PerkinElmer, Inc.; Waltham, MA, USA). In brief, 1–5 mg of accurately weighed sample was enclosed in an aluminum pan and examined across a temperature span of 30–280 °C, using an empty sealed aluminum pan as the reference standard. A nitrogen purge at a flow rate of 20 mL/min was maintained, and the heating rate was kept constant at 5 °C/min.

- X-ray Diffraction Analysis

X-ray diffraction (XRD) characterization was conducted employing a MiniFlex 300/600 diffractometer (Rigaku Corporation, Tokyo, Japan) to measure the crystallin nature of NCMPs. The scanning rate was 10°/min and a diffraction angle (2θ) of 10° to 80°. X-ray diffraction was performed using Copper Kα radiation operating at 35 kV and 15 mA.

- Fourier Transform Infrared Spectroscopy

The Fourier Transform Infrared Spectroscopy (FTIR) characterization was conducted to detect potential chemical interaction between OSI and polymers or among NCMPs excipients. Spectra of OSI, PLGA, chitosan, L-leucine and PLGA-C NCMPs were recorded via Spectrum 100 FTIR spectrometer (Perkin Elmer, Waltham, MA, USA). The measurements were collected within a spectral range of 650–4000 cm^−1^ at a resolution of 4 cm^−1^.

- Moisture content

A thermogravimetric analyzer instrument (PerkinElmer, Waltham, MA, USA) was employed to determine the moisture content of NCMPs powder over a temperature range of 25–500 °C at a heating rate of 10 °C/min in nitrogen gas. NCMPs powder (5 mg) was loaded in an open platinum TGA pan, and moisture loss arising from water evaporation was analyzed between 25 and 120 °C using the Universal Analysis 2000 system.

### 2.6. In Vitro Aerosolization Study

The NCMPs’ pulmonary deposition were studied using the next-generation impactor (NGI, Copley Scientific Ltd., Nottingham, UK) following the procedure of the United States Pharmacopeia [28]. To reduce particle entrainment, a thin film of 1% Tween-80 in methanol (*w*/*v*) solution was applied to each plate, and the methanol was evaporated in a fume hood before measurement. The NCMPs formulations (n = 3) were loaded into 2–5 hydroxypropyl methylcellulose (size 3). Each capsule corresponds to 10–15 mg of spray-dried powder. The capsule was aerosolized using a Cyclohaler (Teva Pharma, Castleford, UK) and delivered into NGI. Prior to inhalation, the capsules were punctured with the Cyclohaler’s actuator. During the sample run, the airflow was maintained at 60 L/min for 4 s employing an HCP5 vacuum pump (Copley Scientific Ltd., Nottingham, UK) coupled with a TPK 2000 critical flow controller (Copley Scientific Ltd., Nottingham, UK). The samples were collected using 0.1% orthophosphoric acid and acetonitrile mixture (20:80) to extract the OSI from the polymer, which was then analyzed by HPLC as mentioned above. After quantitation of OSI in the remaining powder in the capsule and the device, as well as from the stages of the NGI, the total recovery of OSI was expressed relative to the nominal loaded dose of OSI in the capsule. OSI recovery was quantified to confirm that each experiment met acceptable performance criteria. Aerosolization efficiency of each formulation was determined for OSI in terms of mass median aerodynamic diameter (MMAD) and fine particle fraction% (FPF%). Emitted fraction (EF%) was calculated as percentage of OSI recovered in the NGI relative to the overall OSI recovery. The fine particle fraction (FPF%) was calculated as the percentage of the recovered dose corresponding to particles with aerodynamic diameters under 4.5 µm deposited in the NGI. Linear regression was applied to the log-normal plot depicting cumulative OSI fraction against the cut-off diameter for each NGI stage. Based on OSI recovery, the MMAD was determined to be the midpoint in particle size.

### 2.7. In Vitro Drug Release and Kinetics Study

NCMPs equivalent to 90 µg of OSI were weighed precisely and dispersed in 7.0 mL phosphate-buffered saline containing 1% polysorbate 80, adjusted to pH 7.4, then incubated at 37.0 ± 0.5 °C in a water bath at a constant speed (100 rpm) and oscillated away from light. At each predetermined measuring point (1, 2, 4, 6, 8 and 24 h), the supernatant (0.5 mL) was collected by centrifugation, and 120 µL centrifuged supernatants were used for HPLC analysis. Then, NCMPs were resuspended in a fresh medium and continued to be incubated. The release curves of PLGA NCMPs and PLGA-C NCMPs were plotted as cumulative release percentages.

To provide an insight into release behavior of OSI release from NCMPs, a model-dependent technique was used by fitting the release data to the following kinetic models: zero-order kinetic (Equation (5)), first-order kinetic (Equation (6)) and Higuchi model (Equation (7)).

***Q_t_*** = ***Kt*** (5)

***ln Q_t_*** = ***ln Q*_0_ − *Kt*** (6)

***Qt*** = ***Kt*^1/2^** (7)

where Q_t_ is the percentage of drug release at time t, Q_0_ is the initial amount of drug in the formulation and K is the kinetic constant. The model with the highest correlation coefficient (R2) was selected to describe the OSI release mechanism from NCMPs. To further validate the release mechanism, the Korsmeyer–Peppas model (***m_t_/m_∞_ = kt^n^***) was applied. In this model, k represents the release rate constant, and *n* (diffusional exponent) was obtained from the slope of the logarithmic plot of the fraction of drug released (***m_t_/m_∞_***) versus release time (t). For spherical particles, when 0.43 < *n* < 0.85, diffusion and polymer relaxation/swelling or erosion is the dominant mechanism of drug release (anomalous transport/non-Fickian diffusion). If *n* value is 0.43 or less, the drug release is mainly governed by the diffusion of the drug through polymer matrix (Fickian diffusion/Case I transport). If *n* value is close to 0.85, the drug release is mainly governed by polymer erosion and/or relaxation (Case II transport) and when *n* = 1, the release is zero order [29].

### 2.8. Stability Study

The formulations were stored in a desiccator in a cabinet in glass containers at 25 °C in absence of light for three months. The stability of the NCMPs was evaluated by screening the morphology, particle size and solid state as described above.

### 2.9. In Vitro Antitumor Effect Studies

#### 2.9.1. Cell Culture

Human non-small cell lung cancer (NSCLC) cell line (A-549) was maintained in DMEM media supplemented with 10% of heat-inactivated FBS, 100 µg/mL streptomycin, and 100 units/mL of penicillin, under a humidified 5% CO_2_ atmosphere at 37 °C.

#### 2.9.2. Cytotoxicity Assay

Cytotoxicity testing of PLGA NPs, PLGA-C NPs and free drug (OSI) was conducted in vitro via the sulforhodamine B (SRB) assay. For this assay, A-549 cell suspension (5 × 10^3^ cells) was seeded in 96-well plates using 100 μL of cell suspension and incubated in complete medium for 24 h. Subsequently, cells were treated with an additional 100 μL of medium containing OSI or formulations at various concentrations, and DMSO was used as a positive control. After a 72 h treatment, cell fixation was performed via substituting medium with 150 μL of 10% trichloroacetic acid (TCA), and cells were incubated at 4 °C for one hour. Following TCA removal, the cells were rinsed with distilled water (five times). Subsequently, a volume of 70 μL of SRB solution (0.4%, *w*/*v*) was employed and kept for 10 min at room temperature in the dark. Plates were then washed three times with 1% acetic acid and allowed to air-dry overnight. To solubilize the protein-bound SRB stain, a volume of 150 μL of 10 mM TRIS was dispensed into each well. Measurements of absorbance at 540 nm were performed employing an Infinite F50 microplate reader (TECAN, Zurich, Switzerland). Results were reported as percentages based on the absorbance ratio between treated wells and untreated controls. To compute the half inhibitory concentration (IC50), the SigmaPlot^®^ 12.0 software (Grafiti LLC, San Jose, CA, USA) was used to perform the analysis. Using the same method, the influence of the blank formulation on cell viability was examined to ensure safety.

#### 2.9.3. Cell Migration Assay

The in vitro cell migration assay was performed using the wound healing assay to evaluate the migratory potential of the cancer cells. A549 cells were plated at density 2 × 10^5^/well onto a coated 12-well plate and cultured overnight to allow adherence and form a confluent monolayer. The following day, scratches were made on the confluent cell monolayer using a sterile 200 µL pipette tip, and the wells were subsequently washed with PBS. Control wells received fresh medium, while treatment wells were supplemented with medium containing OSI or PLGA-C NPs. Images were captured at 0, 24, 48, 72, and 96 h using an inverted microscope with plates incubated at 37 °C and 5% CO_2_ between time points. Light microscopy images were captured using an Olympus CKX41 inverted microscope equipped with phase-contrast optics. Migration was quantified as the percentage reduction in wound area over time (Equation (8)).

(8) $$\mathrm{Wound}\;\mathrm{closure}\%=\frac{A_{t=0}-A_{t=h}}{A_{t=0}}\times 100$$

A_t=0h_ represents the mean wound area recorded at time zero (immediately post-scratch), whereas A_t=Δh_ denotes the mean wound area recorded h hours post-scratch.

#### 2.9.4. Cell Cycle Assay

After treating A549 cells with OSI and PLGA-C NPs at IC50 for 72 h, the treated cells were harvested through trypsinization. Subsequently, the collected cells were subjected to two consecutive washes using ice-cold PBS with a pH of 7.4 to ensure the removal of residual media and reagents. The resulting cell pellet was then carefully resuspended in 2 mL of 60% ice-cold ethanol, followed by fixation at 4 °C for a period of one hour to preserve cellular integrity. Then, the cells underwent two additional washes with PBS to eliminate any remaining ethanol. The washed cells were then resuspended in 1 mL of PBS supplemented with 50 µg/mL RNase A to degrade RNA and 10 µg/mL propidium iodide to stain DNA. This prepared suspension was incubated in complete darkness at 37 °C for 20 min to allow optimal staining and enzymatic activity. Subsequently, the DNA content of the stained cells was analyzed using flow cytometry, specifically employing an ACEA Novocyte^TM^ flow cytometer (ACEA Biosciences Inc., San Diego, CA, USA) CONFIGURED FOR FL2 detection with excitation/emission wavelengths of 535/617 nm. For each individual sample, a total of 12,000 cellular events were recorded to ensure statistical reliability. The distribution of cells across different phases of the cell cycle was then determined using ACEA NovoExpress™ software version 1.6.2, providing detailed insights into cell cycle dynamics following treatment.

### 2.10. Statistical Analysis

The statistical evaluation of the experimental data was performed using Minitab^®^ 19 Statistical Software, which provided a comprehensive platform for conducting the required analyses. To determine whether significant differences existed among the experimental groups, a one-way analysis of variance (ANOVA) was applied, and, where appropriate, Student’s *t*-test was employed to compare individual group means. For all statistical tests, a *p*-value of less than 0.05 was considered indicative of statistical significance. All numerical results are recorded as the average value accompanied by the corresponding standard deviation, based on three independent replicates.

## 3. Results and Discussion

### 3.1. Characterization of Nanoparticles

Table 1 displays physicochemical characterization of OSI-loaded NPs. Both PLGA NPs and PLGA-C NPs formulations had particle sizes smaller than 200 nm, with a comparatively uniform distribution (PDI < 0.3). Previous research found that chitosan adsorption onto PLGA nanoparticle surfaces is attributed to the surface heterogeneity of the nanoparticles and the cationic nature of chitosan. Nanoparticles coated with chitosan typically exhibit greater particle sizes. However, after coating, the particle size was reduced significantly (*p* < 0.05) in this study. The low molecular weight chitosan coated the surface of the PLGA NPs, filled the micropores, and caused charge compression, resulting in a smaller size [24]. It has been revealed that the particles with size less than 260 nm exhibit limited phagocytosis within alveolar regions and not subject to rapid mucociliary clearance, thus helping to avoid the lung-clearance mechanisms and ensure prolonged retention of nanoparticles at the tumor site [30].

**Figure 1:**
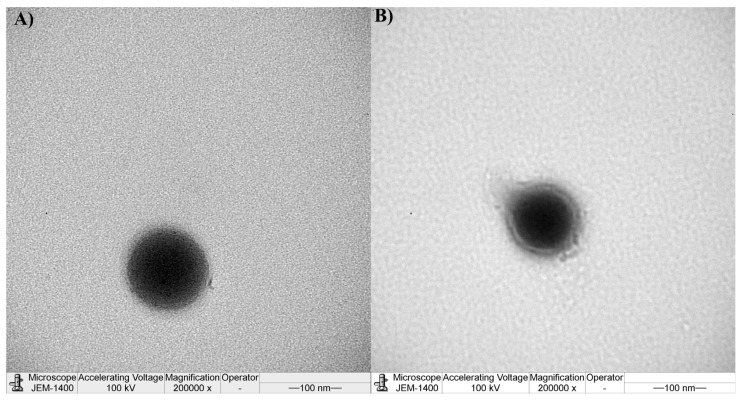
Transmission electron microscopy image: (**A**) PLGA nanoparticles and (**B**) PLGA-C nanoparticles.

The TEM analysis revealed that PLGA and PLGA-C NPs exhibited a uniform spherical morphology (Figure 1). PLGA-C NPs showed a compact polymeric core enveloped by a uniformly distributed chitosan layer. Consistent with size analysis, PLGA-C NPs were smaller than PLGA NPs.

The charge of the PLGA NPs was evaluated by zeta potential analysis to be negative (Table 1). Carboxylic end groups within the PLGA polymer are anticipated to confer a negative charge on the surface of PLGA NPs. The zeta potential increased significantly after coating with chitosan to be positive (Table 1). The increase in charge indicated that chitosan was successfully coated on the surface of PLGA-C NPs. This effect may result from amino groups present on the nanoparticle surface [16].

PLGA NPs and PLGA-C NPs displayed high EE% (Table 1). This may be due to the intrinsic hydrophobic properties of OSI, which allow OSI to enter the hydrophobic interior of NPs [31]. Moreover, EE% changed insignificantly (*p* > 0.05) on the addition of chitosan since chitosan was added to the aqueous phase. Therefore, it is unlikely to have influenced the viscosity of the organic phase or the diffusion of the drug into the aqueous phase [29]. Furthermore, high EE% is expected to reduce material loss, improve particle production, and lower manufacturing costs. The actual drug loading of PLGA NPs was determined to be 39.14 ± 0.06 µg/mg and it decreased significantly (*p* < 0.05) after coating with chitosan to 12.76 ± 0.01 µg/mg. This was expected as when the total mass of NPs increased, the drug loading value decreased.

### 3.2. Characterization of Nanocomposites Microparticles

**Figure 2:**
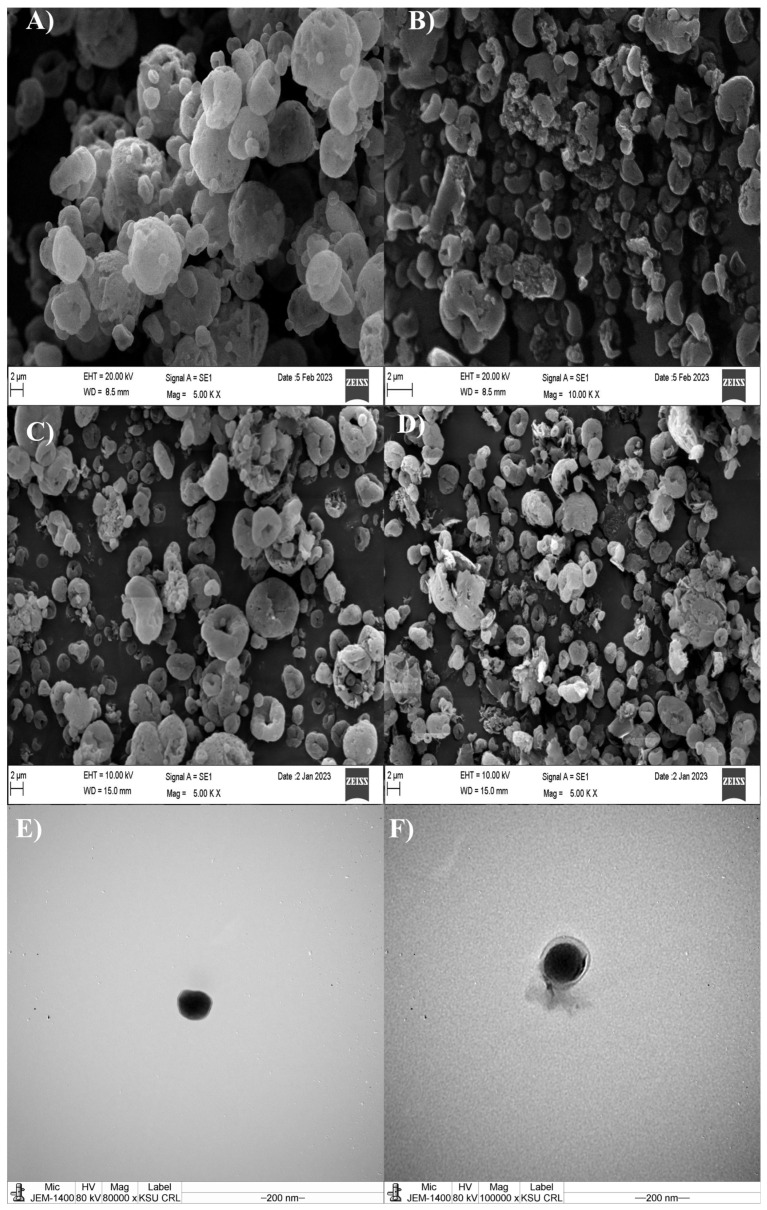
Scanning electron microscopy image of freshly prepared formulations (**A**) PLGA NCMPs and (**B**) PLGA-C NCMPs, and after three months of storage at 25 °C: (**C**) PLGA NCMPs, and (**D**) PLGA-C NCMPs. Scanning electron microscopy image of nanoparticles after redispersion of NCMPs (**E**) PLGA NPs and (**F**) PLGA-C NPs. The scale bar represents 2 μm.

Figure 2A,B represents the SEM photomicrographs of NCMPs. The shape and surface were irregularly dimpled and corrugated NCMPs. This has been consistent with the known spray-drying behavior of leucine due to the vapor pressure generated during water removal in the spray-drying process, suggesting that leucine contributed to the morphology of NCMP and improved aerosolization [20]. The SEM analysis (Figure 2A–D) revealed that the NCMPs exhibited particle sizes of 3.99 ± 0.1 µm for PLGA NCMPs and 2.94 ± 0.1 µm for PLGA-C NCMPs, which meet the geometric size criteria for inhalation formulations. The yield% of PLGA NCMPs and PLGA-C NCMPs were 57.61 ± 4.5 and 41.72 ± 3.1%, respectively.

It is worth mentioning that when NCMPs re-dispersed, the size of the NPs returned to approximately their original size before spray drying, with a slight increase (Figure 2E,F). Slight particle size increase after spray-drying can be attributed to interparticle cohesiveness primarily driven by Van der Waals interactions, capillary forces, and nanoparticle aggregation under the operational conditions of spray-drying. After reconstitution, the particle size was 211.97 ± 93.8 nm for PLGA NPs and 181.64 ± 54.1 nm for PLGA-C NPs. The calculated re-dispersibility index (RdI) further supports this observation, with RdI values of 1.27 for PLGA NPs and 1.25 for PLGA-C NPs, indicating acceptable redispersion within the expected range (0.7–1.3) [27].

### 3.3. Solid-State Characterization of Nanocomposites Microparticles

#### 3.3.1. Differential Scanning Calorimetry

**Figure 3:**
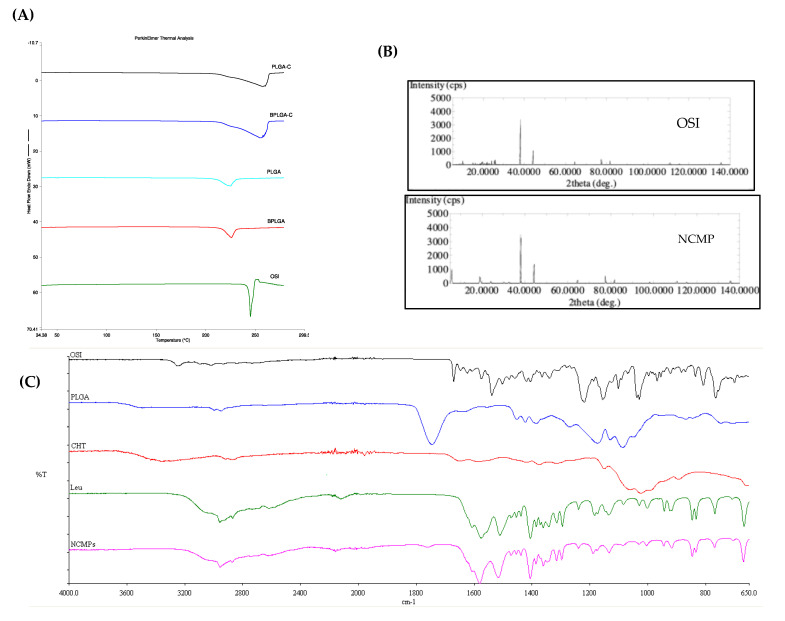
(**A**) The DSC thermograms of osimertinib-loaded PLGA-C NCMPs (PLGA-C), Blank PLGA-C NCMPs (BPLGA-C), osimertinib-loaded PLGA NCMPs (PLGA), Blank PLGA NCMPs (BPLGA), and pure osimertinib (OSI). (**B**) The XRD diffractograms of pure osimertinib (OSI) and osimertinib loaded PLGA-C NCMPs (PLGA-C NCMPs), (**C**). The FTIR spectrum of pure osimertinib (OSI), PLGA, chitosan (CHT), L-leucine (Leu) and osimertinib-loaded PLGA-C NCMPs (NCMPs).

A comparative thermogram of pure OSI, PLGA NCMPs, and PLGA-C NCMPs is shown in Figure 3A. The thermogram of pure OSI showed sharp endothermic peak at 245.13 °C due to its melting transition confirming its crystalline nature [32]. The absence of a sharp peak in PLGA NCMPs and PLGA-C NCMPs indicated complete drug encapsulation in the NCMPs. Moreover, no extra peak was recorded, indicating that there is no interaction between OSI and the excipients.

#### 3.3.2. Powder X-Ray Diffraction (PXRD)

To evaluate the crystalline nature of the NCMPs and confirm OSI encapsulation, PXRD analyses were performed. Due to its crystalline structure, OSI showed intense sharp peaks at 2θ values of 24.14°, 24.78°, 25.56°, and 27.66° in XRD spectra (Figure 3B). The OSI characteristics peaks were absent in the XRD spectra of NCMPs as shown in Figure 3B, confirming OSI encapsulation and suggesting a consistent result with that of DSC.

#### 3.3.3. Fourier Transform Infrared Spectroscopy (FTIR)

The interaction between OSI and excipients was characterized by FTIR. The FTIR spectra of OSI, PLGA, chitosan, L-leucine and PLGA-C NCMPs are presented in Figure 3C. OSI has characteristic peaks at about 3247 cm^−1^ due to N-H stretching of the secondary amine group, and 1670 cm^−1^ due to C=O stretching of the amide group. There was a rich fingerprint region below 1646 cm^−1^, showing various bending and stretching vibrations from aromatic rings, amines, and ether groups [33].

The FT-IR spectra of PLGA exhibited characteristics peaks, including a C=O stretching band at 1745 cm^−1^, a C(=O)-O stretching band at 1173 cm^−1^, and an O-C-C stretching band at 1086 cm^−1^, confirming the presence of ester functional group typical of PLGA. Additional bands observed at 2946, 1423 and 1383 cm^−1^ corresponding to C-H stretching, and asymmetric and symmetric C-H bending vibrations, respectively [34].

Chitosan FT-IR spectrum displayed a peak attributed to the presence of polysaccharide backbone at1024 cm^−1^. Peaks at 3351 cm^−1^, 1586 cm^−1^, and 1375 cm^−1^ can be assigned to stretching vibration of hydroxyl, amine group and residual acetyl groups, respectively [16].

L-leucine FT-IR spectrum displayed a peak at 2956 cm^−1^ due to its amino acid group stretching. The observed bands at 1575 cm^−1^ are attributed to carbonyl group stretching, which is further supported by the presence of a medium-intensity band at 1404 cm^−1^ corresponding to COO^−^ asymmetric vibration. The observed asymmetric (1607 cm^−1^) and symmetric (1511 cm^−1^) are assigned to the bending vibration of N-H band [34].

The FTIR spectra of the spray-dried formulations (NCMPs) with L-leucine exhibited no new peaks or significant spectral shifts, indicating no major chemical bond formation or degradation products. The L-leucine peaks are clearly visible and prominent, especially the methyl group stretching and carbonyl group stretching, confirming the embedding of the nanoparticles into L-leucine and the successful formation of NCMPs.

#### 3.3.4. Thermo-Gravimetric Analysis (TGA)

A high residual moisture content enhances particle aggregation, which changes the particle size distribution and the aerosol performance [35]. To verify the effectiveness of the spray-drying method, TGA was used to evaluate the residual moisture content of the NCMPs. The moisture content of the formulation is indicated by mass loss as a function of temperature between 20 and 130 °C. NCMPs lost approximately 0.41 ± 0.003% of their respective masses at temperatures between 25 and 130 °C, indicating the drying employed during the spray drying process was efficient. This can be attributed to the hydrophobic L-leucine [36].

### 3.4. In Vitro Pulmonary Deposition

**Figure 4:**
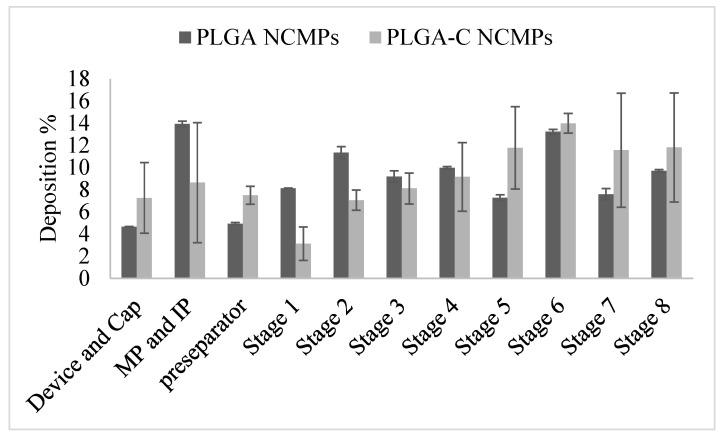
In vitro aerosol deposition profile of Osimertinib loaded NCMPs expressed as % deposition at each stage. MP and IP refer to mouthpiece and induction port. Data represents mean ± SD (n = 3).

The aerodynamic properties of inhalable NCMPs were tested using NGI to evaluate their pulmonary deposition across different lung regions. Figure 4 illustrates the in vitro aerosol deposition profile of the drug across the NGI stages. According to the log-probability analysis, the MMAD, which represents the median aerodynamic diameter by mass, ranged between 1 and 2 μm (Table 2). The MMAD values obtained fell well within the acceptable range of 1–5 μm, which is required for effective aerosol deposition in the respiratory tract [37]. Moreover, the formulations displayed good value of FPF % (Table 2), exhibiting aerosolization properties suitable for the central and even peripheral regions of the lungs [38]. There was no significant difference in deposition for both NCMPs formulations; therefore, the addition of chitosan had no effect on the aerosolization properties [39].

### 3.5. In Vitro Release and Kinetics Study

**Figure 5:**
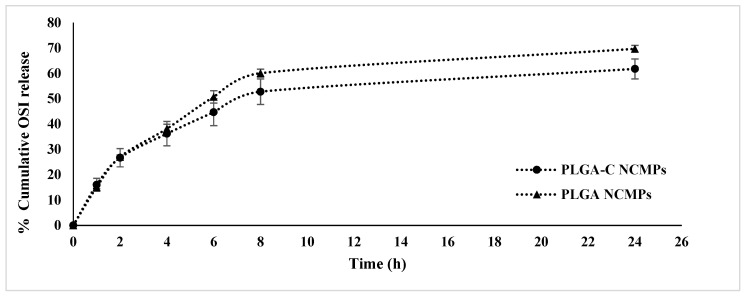
In vitro release of osimertinib loaded PLGA and PLGA-C NCMPs. Data represents mean ± SD (n = 3).

To evaluate the sustained-release properties of NCMPs, the cumulative drug release was measured in PBS including 1% polysorbate 80, adjusted to pH 7.4 and maintained at 37 °C (Figure 5). The OSI release from NCMPs showed two different phases. The PLGA NCMPs and PLGA-C NCMPs exhibited a 15% burst release within the first hour. The initial burst release from NCMPs may be attributed to the rapid desorption of OSI from or near the NCMPs surface, combined with the increased diffusion of the medium across the surface. The slower release phase is likely attributed to OSI being encapsulated within the PLGA polymeric matrix, where diffusion occurs gradually because the hydrophobic nature of PLGA limits water penetration into the matrix. Moreover, this may be caused by the extended diffusion pathway of OSI molecules enclosed within the PLGA matrix core, with subsequently slow bioerosion of the polymer. The PLGA-C NCMPs modified with chitosan displayed a slower release rate after 24 h. This effect might result from the outer chitosan layer that reduced hydrolysis of the NPs structure. Almost 69.68 ± 1.33 and 61.76 ± 3.9% of the OSI was released from the PLGA NCMPs and PLGA-C NCMPs, respectively, within a period of 24 h. The slow and sustained release pattern observed is expected to contribute to prolonged tumor suppression [18,39].

Three mathematical models were used to study the drug-release mechanism of NCMPs, and the fitting results are listed in Table 3. The best fit was selected based on the R^2^ values of OSI release kinetics. It was observed that the release of OSI from PLGA NCMPs and PLGA-C NCMPs is best described by the Higuchi model (R^2^ > 0.9) suggesting diffusion-controlled drug release. To gain deeper insight into the diffusion process, the Korsmeyer–Peppas model was employed. A non-Fickian (anomalous) release mechanism (n = 0.49) was identified for PLGA NCMPs, indicating that OSI release was driven by simultaneous diffusion and polymer erosion processes. Fickian diffusion (Case I Transport) could be suggested to control OSI release from PLGA-C NCMPs since n = 0.43. This finding suggests that the chitosan coating might be very thin or dense, minimizing its contribution to polymer relaxation/swelling-driven release. Furthermore, at the pH (7.4) of the release medium, the swelling of chitosan is limited due to deprotonation of amino groups reducing its solubility and swelling [40,41].

### 3.6. Stability Study

Moisture and storage time are one of the common factors that can influence the physicochemical stability of dry powder formulations. These conditions may induce changes in the solid-state properties, leading to increased particles aggregation. Moisture may enhance drug molecule mobility, leading to the crystallization of amorphous dry powder formulations. Furthermore, because solid-state transformation can alter the therapeutic efficacy of the formulation, it is essential to maintain the solid-state integrity of both the drug and excipients throughout the manufacturing process and storage [42]. As a result, the evaluation of the solid-state behavior during storage is crucial. In this study, to investigate the effect of residual moisture and storage time, a stability study was performed. SEM, particles size, DSC, and TGA analyses were performed on all samples post-storage. Storage of NCMPs was carried out at 25 °C for a period of three months.

**Figure 6:**
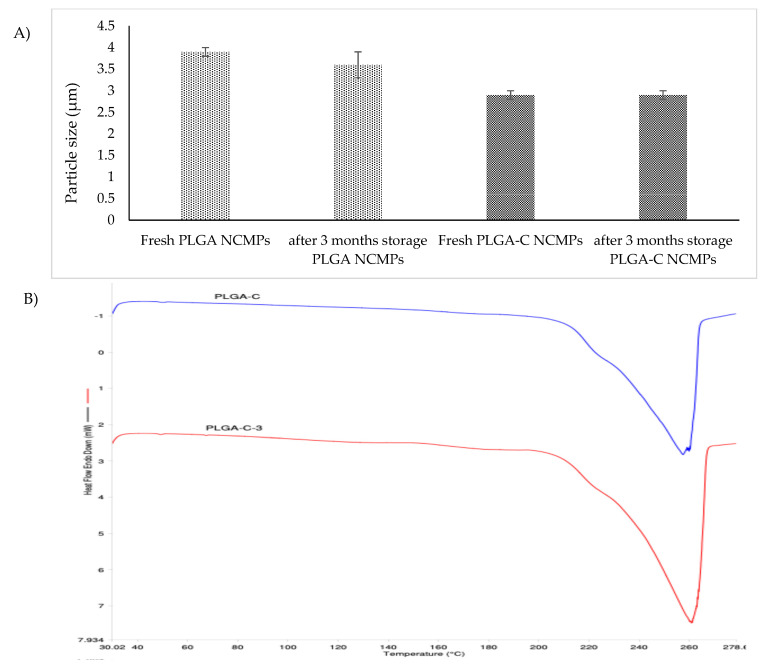
(**A**) Particle size of freshly prepared NCMPs and after three months storage at 25 °C. Data represents mean ± SD (n = 3). (**B**) DSC thermograms of PLGA-C NCMPs after three months storage at 25 °C.

The morphologies of NCMPs stored at 25 °C for three months were monitored, NCMPs retained their shape and surface morphology with no major morphological changes observed indicating good physical stability (Figure 2E,F). In accordance with SEM images, NCMPs particle size did not change significantly after three months of storage at 25 °C (Figure 6A).

The similar melting transition properties of fresh NCMPs and after 3 months of storage show the NCMPs remained unaffected during storage (Figure 6B). Moreover, OSI endothermic peaks were not present in NCMPs after 3 months of storage, which indicates that no crystalline OSI was found in NCMPs after 3 months of storage.

To determine the residual moisture content during storage, TGA of dry powder formulation was also performed. The percent weight change between 20 and 130 °C indicates the removal of water content from the formulation. The TGA measurements revealed that the thermal mass loss was below 0.5 ± 0.001% after three months of storage, verifying moisture absence and maintaining the stability of the formulation.

### 3.7. Evaluation of the Safety of Nanoparticles In Vitro

**Figure 7:**
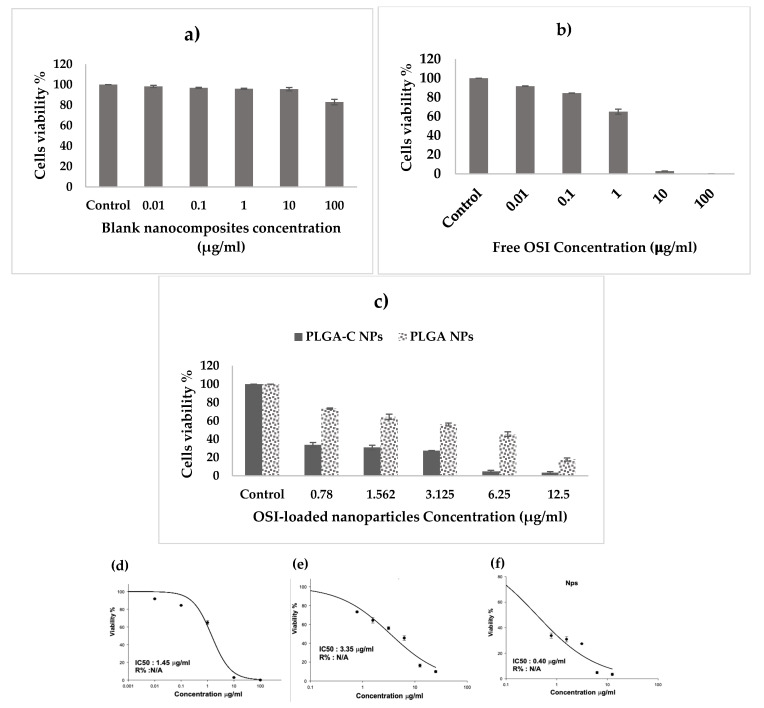
Cytotoxic effect of osimertinib in various formulations on A-549 cell line. A-549 cells were treated with (**a**) blank nanoparticles, (**b**) different concentration of free osimertinib, (**c**) osimertinib-loaded nanoparticles (absolute concentrations applied refer to concentration of osimertinib) for 72 h. Cells’ viability was measured by SRB assay. Data represents mean ± SD (n = 3). Dose–response curves used to calculate IC_50_ values for A549 cells after treatment with (**d**) free osimertinib, (**e**) osimertinib loaded PLGA NPs, (**f**) osimertinib loaded PLGA-C NPs (absolute concentrations applied refer to concentration of osimertinib) for 72 h of incubation using the SRB assay.

A-549 cell viability was determined through the SRB assay to evaluate the cellular safety of blank formulation. Figure 7a shows that the cell survival rate decreased with the increase in the formulation concentration. However, the cell survival percentage was still higher than 90%. The results demonstrated that there was no apparent cytotoxicity by any concentration of blank formulation on the survival percent of A-549 cells, which makes it potentially nontoxic, highly biocompatible, and safe.

### 3.8. Antitumor Effects In Vitro

The in vitro antitumor effect of the free OSI and formulations was studied in the NSCLC cell line, A-549 (Figure 7b,c). The free OSI demonstrated an IC50 value of 1.45 ± 0.12 µg/mL, thereby indicating the high potency of the drug on the A-549 cells. The encapsulation of OSI in PLGA-C NPs led to a 3.6-fold decrease in IC50 (IC50 of 0.4 ± 0.10 µg/mL) versus free OSI (*p* < 0.05). Furthermore, there was an 8.34-fold reduction in the IC50 value for the PLGA-C NPs compared to the PLGA NPs (IC50 of 3.35 ± 0.31 µg/mL, *p* < 0.05). OSI encapsulated in PLGA-C NPs exhibited the lowest IC50, indicating that a significantly reduced drug dose in the NPs achieved high therapeutic efficacy. The improved cytotoxicity of the PLGA-C NPs possibly resulted from improved cellular uptake facilitated by the chitosan coating [43]. The encapsulation of OSI in polymer-based or lipid-based nanoparticles augments its therapeutic effect. For instance, the coating of OSI-loaded PLGA nanoparticles with chitooligosaccharide significantly potentiates the antitumor action of OSI. This strategy enhanced greater cellular uptake and sustained release, resulting in an enhanced aantitumoractivity [16]. Similarly, Kohei Tahara and colleagues demonstrated that chitosan-modified PLGA nanospheres exhibited significantly higher uptake by A549 lung cancer cells compared to unmodified PLGA nanospheres, confirming that chitosan coating improves nanoparticle internalization [43]. Moreover, Raval et al. reported that inhalation therapy using silibinin-loaded, chitosan-coated PLGA/PCL nanoparticles for inhalation therapy resulted in markedly enhanced cytotoxicity and bioavailability against lung cancer cells, primarily due to increased cellular internalization and sustained drug release [16]. These data along with our results suggest that when OSI is encapsulated in a suitable nanoparticle, it will provide improved anantitumorfficacy by promoting cellular uptake and increasing drug concentration within the tumor region. Considering the greater in vitro antitumor efficacy, the PLGA-C NPs was selected for further in vitro studies.

### 3.9. Cell Migration Assay

**Figure 8:**
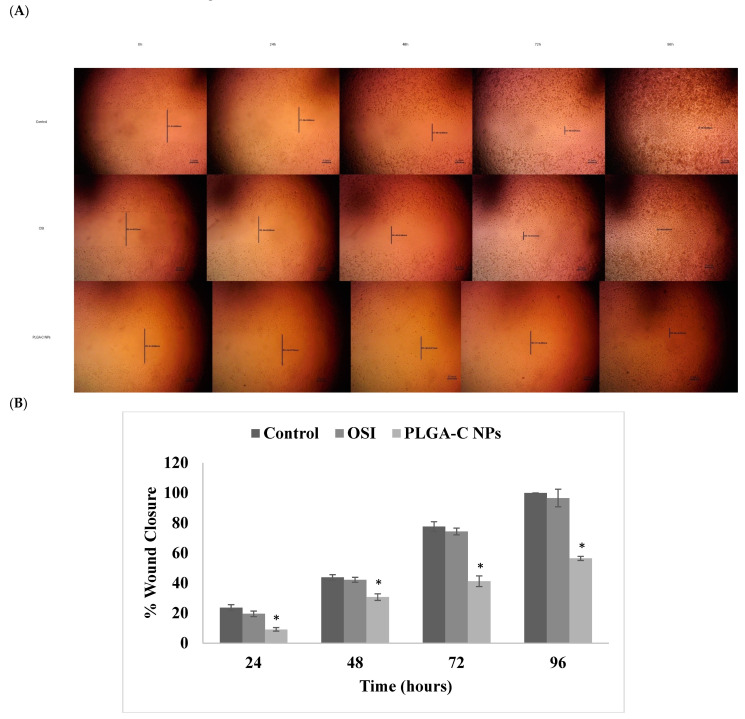
(**A**) Wound healing microscopic images of the wound at 0 h, 24 h, 48 h and 96 h after treatment. Scale bars = 200 μm. (**B**) Quantitative assessment of wound closure in A549 cells treated with normal medium (control), OSI, or PLGA-C NPs for 96 h. Results are expressed as percentage wound closure over time. Data are presented as mean ± SD (n = 3), with (* *p* < 0.05).

It is well known that cancer cells can spread (metastasize) to distal organs such as bone, brain, and liver. When cancer cells metastasize, the disease will be worsened and it significantly increases mortality rate [44]. Thus, it is essential to formulate a treatment that has a cytotoxic effect on cancer cells and inhibits cell migration and metastasis. Therefore, the antimigration properties of free OSI and PLGA-C NPs was evaluated using the wound-healing assay. The effect of OSI and PLGA-C NPs on the A549 cells viability was measured at concentrations up to 100 and 12.5 µg/mL, respectively (Figure 7b,c). At 0.2 µg/mL OSI and PLGA-C NPs showed over 80 and 75% cell viability, respectively. Therefore, A549 cell invasion was tested at a concentration of 0.2 µg/mL to exclude the growth inhibitory effect of OSI or PLGA-C NPs on migration. A wound was created at 0 h and monitored for 96 h following the respective treatments, as shown in Figure 8. Cell migration in treatment groups, i.e., OSI and PLGA-C NPs, was compared to that of the control group. Wound closure (%) was calculated for each group relative to its initial wound area at 0 h. As shown in Figure 8A,B, after 96 h of incubation, wound closure was significantly reduced (*p* < 0.05) in the PLA-C NPs compared to both the control and OSI groups, suggesting a potential inhibitory effect on cell migration. Furthermore, PLGA-C NPs demonstrated 56.45 ± 1.38% wound closure, which is significantly (*p* < 0.05) lower than free OSI 96.62 ± 5.8%, displaying a stronger inhibition effect. This could be attributed to the controlled and prolonged release of OSI from the PLGA-C NPs over 96 h. These findings demonstrate that the PLGA-C NPs have a stronger inhibitory effect on the migratory properties of A549 cells than free OSI. Moreover, this indicated that encapsulating OSI within PLGA-C NPs enhances the therapeutic effect of OSI in suppressing A549 cell migration. Similarly, Payomhom et al. examined chitosan-coated PLGA nanoparticles loaded with ursolic acid on MDA-MB-231 cells, which are characterized by their metastatic and invasive nature. Their findings from the wound-healing assay were consistent, showing that the chitosan-coated PLGA nanoparticles formulation exhibited minimal cell migration [45].

### 3.10. Cell Cycle Assay

**Figure 9:**
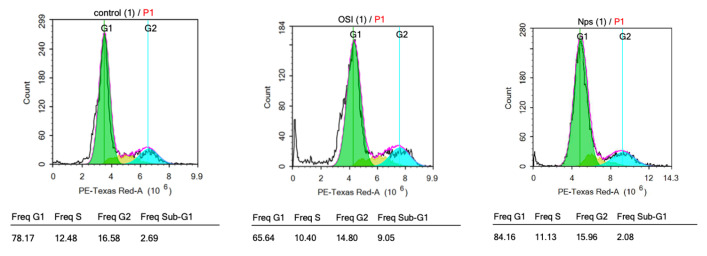
Cell cycle profiles of A-549 cells after no treatment (control) and after treatment with Osimertinib (OSI) and PLGA-C NPs (Nps) at their respective IC50 concentrations for 72 h.

The cell cycle encompasses a sequence of events that culminate in cellular division and replication. Its regulation involves essential processes that are critical for cell survival. The cell cycle analysis was used to evaluate how the OSI encapsulation in PLGA-C NPs affects the progression of lung cancer cells through different phases of the cell cycle (Table 4, Figure 9). OSI exerted growth inhibitory effects on the non-small cell lung carcinoma cell line A549 at its IC50 over 72 h. Flow cytometric analysis revealed that the shift in cell distribution into the G0/G1 phase was augmented by PLGA-C NPs significantly (*p* < 0.05) compared to OSI-treated and untreated control groups. Furthermore, PLGA-C NPs and OSI resulted in a significant reduction (*p* < 0.05) in the proportion of cells in the S phase compared to the control group. This accumulation at the primary cell cycle checkpoint was accompanied by a marked reduction in the percentage of cells entering the S phase, indicating effective inhibition of DNA replication and cell cycle progression. Thereby cell proliferation prevention accompanied by promoting cell death. This suggests that the PLGA-C NPs may facilitate improved cellular uptake and endosomal escape, thereby increasing the intercellular concentration of OSI and enhancing its ability to disrupt the G1/S phase signaling cascade. Furthermore, this potent anti-proliferative effect correlated with antimigration activity (Section 3.9) demonstrated by PLGA-C NPs compared to both the OSI and untreated group. These observations are consistent with a newly published study by Fong et al., which demonstrated the chitosan-coated PLGA NPs enhanced tumor-inhibiting activity and antimigration activity of Stattic, a STAT3 inhibitor, through similar mechanisms of G0/G1 arrest and reduced motility [45]. Furthermore, Nanamiya et al. identified EphB4 as a novel target mediating the EGFR-independent suppressive effects of OSI in NSCLC. Their work showed that OSI induces G0/G1 arrest via activation of the p53-p21 axis and downregulation of Cyclin D1, even in EGFR-knockdown cells [46].

## 4. Conclusions

The formulation of a dry powder inhalable OSI delivery system designed to facilitate tumor-targeted delivery and minimize systemic toxicity is an attractive proposition. In this research, PLGA-C NPs were developed employing emulsification followed by a solvent evaporation process. The PLGA-C NPs exhibited a high drug-loading capacity, small particle size and a positively charged zeta potential. In vitro studies in A-549 cell line revealed enhanced cytotoxicity of PLGA-C NPs, with lower IC50 compared to free OSI. Furthermore, the PLGA-C NPs exhibited superior antimigratory effects and a more pronounced induction of cell cycle arrest relative to free OSI. Furthermore, PLGA-C NPs were assembled into respirable NCMPs through a spray-drying technique, with L-leucine employed as the carrier. Solid-state characterization of NCMPs demonstrated encapsulation of drug in the polymer, with no chemical interaction observed. The NCMPs exhibited the desired aerosolization properties and proposed an alternative approach for inhalation-based administration. OSI release studies showed sustained drug release for 24 h. Stability studies confirmed the physicochemical stability of NCMPs. These successful results for the OSI dry powder formulation as an inhalable NSCLC therapy may promote better therapeutic outcomes with reduced systemic toxicity. One constraint of this study is the lack of in vitro studies applying multiple cell lines and the lack of in vivo experiments to confirm pulmonary delivery, tissue distribution, and pharmacokinetic profiles. Future work will first focus on expanding in vitro investigations to include additional cell lines such as macrophages to better assess nanoparticle uptake and pulmonary targeting, followed by in vivo studies to validate the aerodynamic performance, tissue distribution, and therapeutic potential of the developed NCMPs within the complex pulmonary environment.

## Figures and Tables

**Table 1:** Physicochemical characterization of Osimertinib-loaded nanoparticles. Data represents mean ± SD (n = 3).

Formulation	Particle Size (nm)	Polydispersity Index	Zeta Potential (mV)	Entrapment Efficiency (%)	Drug Loading (µg/mg)
PLGA NPs	167.41 ± 2.6	0.185 ± 0.01	−19.8 ± 0.64	98.18 ± 1.01	39.14 ± 0.06
PLGA-C NPs	145.18 ± 3.0	0.153 ± 0.01	+25.6 ± 0.1	97.10 ± 1.04	12.76 ± 0.01

**Table 2:** Aerosolization properties of Osimertinib loaded NCMPs. Data represents mean ± SD (n = 3).

	PLGA NCMPs	PLGA-C NCMPs
FPF (%)	68.33 ± 0.4	73.48 ± 8.6
EF (%)	95.34 ± 3.3	92.74 ± 3.2
Recovered dose (%)	82.04 ± 2.2	93.2 ± 8.6
MMAD (μm)	1.43 ± 0.18	1.09 ± 0.23

**Table 3:** The Correlation Coefficient (r^2^) of the released osimertinib according to different models’ equations.

Model	Formulation	
	PLGA NCMPs	PLGA-C NCMPs
First order	0.7867	0.7613
Zero order	0.6598	0.6477
Higuchi	0.9009	0.9022
Korsmeyer-Peppas	0.9142	0.9257

**Table 4:** Percentages of A549 cells in each phase of the cell cycle following treatment with OSI and PLGA-C NPs (at IC50 values) for 72 h. Data represents mean ± SD (n = 3).

Formulation	Phase		
	G0/G1	S	G2
**Control**	77.80 ± 0.4	13.48 ± 0.9	16.21 ± 0.7
**OSI**	67.58 ± 2.2	11.43 ± 1.1	15.46 ± 1.7
**PLGA-C NCMPs**	83.83 ± 0.3	8.76 ± 2.4	15.52 ± 0.5

## Data Availability

The original contributions presented in this study are included in the article. Further inquiries can be directed to the corresponding author.
